# Disease Tolerance during Viral-Bacterial Co-Infections

**DOI:** 10.3390/v13122362

**Published:** 2021-11-25

**Authors:** Tarani Kanta Barman, Dennis W. Metzger

**Affiliations:** Department of Immunology and Microbial Disease, Albany Medical College, Albany, NY 12208, USA; barmant@amc.edu

**Keywords:** disease tolerance, co-infection, innate immune cells, cytokines, homeostasis

## Abstract

Disease tolerance has emerged as an alternative way, in addition to host resistance, to survive viral-bacterial co-infections. Disease tolerance plays an important role not in reducing pathogen burden, but in maintaining tissue integrity and controlling organ damage. A common co-infection is the synergy observed between influenza virus and *Streptococcus pneumoniae* that results in superinfection and lethality. Several host cytokines and cells have shown promise in promoting tissue protection and damage control while others induce severe immunopathology leading to high levels of morbidity and mortality. The focus of this review is to describe the host cytokines and innate immune cells that mediate disease tolerance and lead to a return to host homeostasis and ultimately, survival during viral-bacterial co-infection.

## 1. Introduction

**Figure 1:**
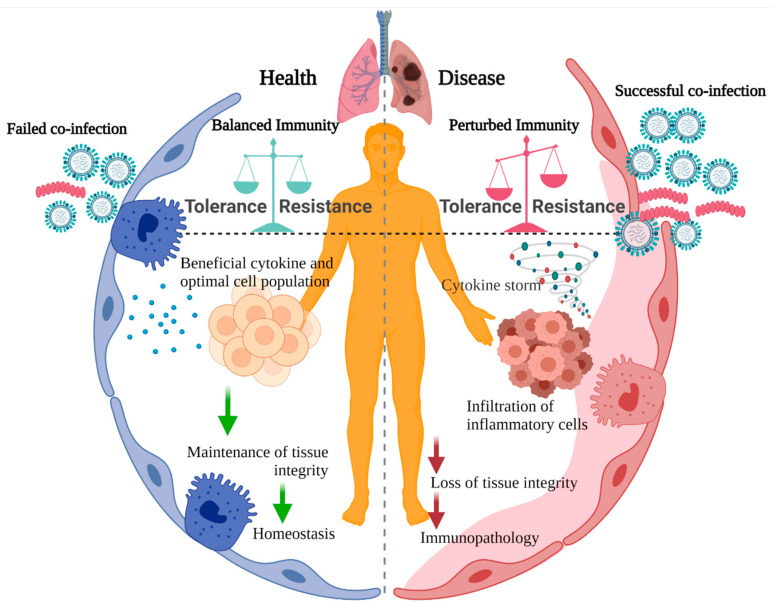
The disease tolerance process and how it impacts the host during co-infection.

Disease tolerance and host resistance are two important arms of innate immunity yet still remain poorly understood. These two complementary defense mechanisms were first recognized by plant scientists [1,2], who classified disease tolerance as a form of resistance [3]. With increased scientific understanding, resistance and tolerance became independent components of the innate immune system. Many elements have been implicated in plant disease tolerance such as the rate of photosynthesis, allocation of nutrients, and genetic traits intrinsic to growth [4,5]. Subsequently, it was also demonstrated that disease tolerance is an evolutionarily conserved immune strategy in all life forms against many types of infection and not directly related to control of pathogen burden [6,7,8]. For example, it has been shown that tolerance defense strategies are present in both Drosophila [9,10] and mammals, including humans [11,12], and allow protection from the deleterious effects of host immunity. Bats are a notable reservoir for numerous viruses such as rabies, Ebola virus, Nipah, severe acute respiratory syndrome coronavirus (SARS-CoV), and Middle East respiratory syndrome coronavirus (MERS-CoV), which are extremely pathogenic in man, SARS-CoV-2 being the most obvious example of this. These viruses are highly virulent in humans, yet bats fail to become sick due to potent disease tolerance mechanisms [13]. The immune system in bats appears to be highly effective at maintaining host homeostasis, a trait developed over 64 million years of evolution [14,15]. In particular, high levels of constitutively expressed type-I interferon (IFN) [16], interferon stimulating genes (ISGs) [17], ABCB1 efflux pump [18], and autophagy [19], as well as increased production of heat shock proteins (HSP) [20], coupled with reduced STING signaling [21] and inflammasome [22] pathways, induces a unique disease tolerance state in bats, allowing the coexistence of many zoonotic viruses, while maintaining tissue integrity following host-pathogen pro-inflammatory interactions. Genetic traits play a vital role in control of disease tolerance [23] and host resistance [24,25,26,27], and both innate and adaptive immune systems contribute to establishing protection from morbidity and mortality through disease tolerance mechanisms [28]. The disease tolerance process and how it impacts the host during co-infection is summarized in Figure 1 (created with BioRender.com (accessed on 28 October 2021)).

## 2. Inflammation in Co-Infection

Co-infection involves illness in a host resulting from multiple pathogens. The major respiratory viral pathogens seen during co-infection are influenza A virus (IAV) and respiratory syncytial virus (RSV), and the bacterial pathogens are *Streptococcus pneumoniae*, *Staphylococcus aureus*, group B Streptococcus and *Haemophilus influenzae* [29,30,31,32].

Inflammation is a protective mechanism against harmful insults from bacteria, viruses or other stimuli, and includes the five cardinal signs of rubor (redness), calor (heat), dolor (pain), tumor (swelling) and *functio laesa* (loss of function). The main goal of inflammation is to eliminate harmful stimuli, clear damaged cells, and heal the tissue [33,34]. However, the immune cells and chemical mediators involved in inflammation can also lead to tissue damage and heightened susceptibility to other infections. The immune cells involved in the inflammatory process are leucocytes, particularly neutrophils [35]. Neutrophils usually reside in the blood and migrate to infected tissues to initiate an inflammatory process. While neutrophils ingest invading bacteria, viruses and cell debris by phagocytosis [36,37], they can also release molecules that damage the epithelial cell barrier. Other cells involved in the inflammatory process are monocytes and lymphocytes. At homeostasis, uninfected lungs contain resting epithelial and resident immune cells at physiological levels, including alveolar macrophages (AM), dendritic cells, natural killer (NK) cells, innate lymphoid cells (ILCs), and B and T cells [38]. Upon infection with IAV, these cells become activated and produce chemokines, which results in recruitment of greater numbers of innate and adaptive immune cells. There are also several cell-derived inflammatory mediators elaborated during the process, such as reactive oxygen species, IL-1, TNF-α, IL-6, and GM-CSF. The pro-inflammatory cytokine, type-II IFN, plays a particularly important role in predisposing the IAV-infected host to secondary bacterial infection by altering macrophage-mediated bacterial clearance [39,40]. In BALB/c mice, increased bacterial burdens correlate with diminished numbers of AM, whereas AM levels are maintained in C57BL/6 mice but the cells have altered phagocytic functions and decreased trafficking between alveoli [39,41]. These effects have been shown to be directly attributable to type-II IFN [39]. IL-10 produced during this process also can regulate lung inflammation and suppress the influx of inflammatory neutrophils [42]. While the entry of inflammatory innate immune cells is likely necessary to resist infection, these infiltrating cells also cause damage to the epithelial barrier integrity and cause alterations in protective innate immunity, leading to pneumonia [38].

Understanding the scientific basis for enhanced susceptibility to bacterial co-infections following influenza remains a significant clinical issue. Influenza-induced inhibition of anti-bacterial innate immunity, especially suppression of AM-mediated bacterial clearance, has been repeatedly implicated by multiple investigators. However, the precise mechanisms responsible for this inhibition are controversial; with some groups reporting an important role for virus-induced type-I IFN [43,44,45,46], possibly associated with decreased CCL2-induced macrophage recruitment [43]. Others have instead defined a critical role of type II IFN [39,40,47,48,49] and direct inhibition of AM-mediated bacterial clearance. In our recent study we observed that influenza co-infection caused IFN-dependent inflammation that facilitated spreading of the colonizing bacteria into the lungs, followed by tissue damage and death [50]. Understanding the pathways responsible for co-infection during influenza has been complicated by several confounding factors. First, the window of susceptibility to bacterial co-infection in humans is typically seen approximately 7–10 days after influenza virus infection, when the viral infection has been nearly totally cleared by the pulmonary immune system. However, some investigators have studied co-infection in animal models at various other times, including bacterial co-infection as early as three days after viral infection [45,51,52], when viral titers and lung inflammation are peaking. Others have examined susceptibility to co-infection several months after influenza [53]. Second, mice have been challenged with various amounts of bacteria and in some cases, very large levels of bacteria that are typically not seen in natural human infection, and which overwhelm the protective AM barrier, leading to active recruitment of highly inflammatory neutrophils in a short period of time [51,54,55]. Third, it is generally believed that humans become co-infected through aspiration of colonizing bacteria [56], yet for technical convenience, many experiments in animal models have involved direct inoculation of bacteria into lungs following influenza [40,44]. Finally, as stated above, AMs show different functional activity that is dependent upon the mouse strain, with depletion of AM during influenza in BALB/c mice versus retention of AMs in viral-infected C57BL/6 mice, but with altered function.

## 3. Cytokines and Disease Tolerance

Many cytokines have already shown promise in tissue protection and damage control although in some cases, they may also induce severe immunopathology that leads to high rates of morbidity and mortality [57,58]. In the following sections, we discuss some important correlates of disease tolerance. We have summarized the tissue protective functions of cytokines in Table 1.

### 3.1. Amphiregulin (AREG)

Recent evidence suggests that AREG is vital for disease tolerance in man and animals. AREG is an epidermal growth factor that is an important mediator of tissue repair at the lung surface during IAV infection [60,93]. Innate cells such as ILC2s, monocytes, basophils, eosinophils, mast cells, neutrophils, and dendritic cells can produce AREG [94,95]. AREG is constitutively produced during homeostasis, but levels increase dramatically following infection and inflammation. AREG binds to the epidermal growth factor receptor and facilitates tissue healing through differentiation and proliferation of epithelial cells [59,96,97,98]. In IAV-infected *Rag1*^−/−^ mice, AREG supports the regeneration of the pulmonary epithelium, and enhances tissue integrity and survival of IAV-infected mice [60].

Mice previously infected with IAV quickly succumb to normally sublethal levels of bacteria during the viral infection recovery stage [61]. This synergistic co-infection is not due to increased viral levels but to increased immunopathology and impaired tissue integrity. Co-infected mice display necrosis of the respiratory epithelial cell layer and genes involved with tissue healing are notably suppressed compared to those in mono-infected animals [61]. Interestingly, IAV-legionella co-infected mice were rescued by administration of AREG into the lungs without reducing pathogen loads [61]. In response to IAV, lung ILC2s can secrete AREG, which promotes epithelial cell proliferation and tissue regeneration following virus-induced tissue damage [95]. Our laboratory demonstrated that mice lacking type-II IFN exhibited elevated levels of AREG in the bronchoalveolar lavage (BAL) following IAV infection along with the increased production of IL-5 which was crucial for improved survival [71]. A similar observation was noted during fungal infection of mice. Treatment with AREG enhanced epithelial repair and increased survival of Dectin-1-deficient hosts compared to wild-type mice [62]. Many bacterial infections such as those caused by *Shigella flexneri*, enterohemorrhagic *Escherichia coli*, *Helicobacter pylori* and *Neisseria gonorrhoeae* also stimulate transcriptional upregulation of AREG in host cells [67,68,69,70]. The severe pathology and tissue damage seen in many parasitic worm or helminth infections can be similarly alleviated by specialized immune strategies [63,64]. Type 2 immunity, including AREG, in particular plays an important role in clearance of these pathogens, healing of damaged tissues, and suppression of inflammation [65]. AREG-deficient mice exhibit delayed clearance of the parasite *Trichuris muris*, which correlates with diminished proliferation of colonic epithelial cells compared to infected wild-type mice [66]. Improved proliferation of epithelial cells in infected wild-type mice is dependent on CD4^+^ T cells [66] indicating epithelial to immune cell communication.

### 3.2. Interleukin-5 (IL-5)

Interleukin-5 (IL-5) is a lineage-specific cytokine that increases eosinopoiesis and plays an important role in diseases that are associated with greater levels of eosinophils, such as asthma [71,72]. IL-3, IL-4 and GM-CSF genes are closely linked to the IL-5 gene locus [99,100,101] and IL-5 expression is controlled by several transcription factors including GATA3 [102]. IL-5 is expressed by an array of cells such as eosinophils, Th2 cells, mast cells, non-hematopoietic cells, NK and natural killer T cells (NKT), Reed Sternberg cells, EBV-transformed cells and ILC2s in the mouse [103,104,105].

Recent evidence has shown that in IAV infection, there can be ample production of lung IL-5 especially in the absence of IFN-γ, which stimulates the progressive recruitment of eosinophils, particularly in the later stage of infection, i.e., during the viral clearance and host recovery phase [71,72]. This IL-5 is mainly produced by a small number of ILC2s in the IAV-infected lung, beginning 5–7 days post-infection, which also corresponds to induction of IAV-specific lung adaptive immunity. Both ILC2 numbers and their ability to produce IL-5 peak following virus clearance and during the 8 to 10 days post-infection recovery phase. This increase in IL-5 production by ILC2s is in part stimulated by IL-33 produced by epithelial cells, AMs and NKT cells infiltrating the IAV-infected lung [106,107,108]. Type-II IFN appears to play a major role in suppressing ILC2-mediated protection, namely in restricting the production of IL-5. Inhibition of IL-5 with neutralizing antibodies in type-II IFN-deficient mice resulted in reduced survival, indicating that enhanced protection in the absence of type-II IFN was dependent on IL-5 and possibly increased eosinophilia [71,72]. These results established a role for ILC2s in promoting disease resistance and tissue integrity against influenza.

### 3.3. Interleukin-22 (IL-22)

IL-22 is an alpha-helical cytokine expressed by immune cells at sites of inflammation. It acts on nonhematopoietic cells at mucosal surfaces, such as epithelial and stromal cells [109]. IL-22 is produced by T cells, NKT cells, ILCs, neutrophils and macrophages [110]. IL-22 can have both healing and pathological roles during infection [109,111,112]. Reduction of inflammation by IL-22 is similar to that mediated by IL-10 during IAV infection and protects against secondary bacterial infection [73]. IL-22 alters several genes involved in epithelial growth and proliferation during IAV infection and treatment of mice with IL-22 improves tissue integrity [74]. IL-22 is also involved in regeneration and repair of the airways following influenza and of intestinal epithelium during ulcerative colitis [75,76,77]. An increased understanding of IL-22 involvement in disease tolerance pathways would help in developing new therapies to enhance tissue repair mechanisms.

### 3.4. Transforming Growth Factor Beta (TGF-β)

TGF-β has three mammalian isoforms, TGF-β1, TGF-β2, and TGF-β3. Among these, TGF-β1 is responsible for maintaining host homeostasis. Because of this, knocking out TGF-β1 in mice results in fatal inflammatory disease and death [113,114,115]. Some of the critical functions of TGF-β1 include inhibition of maturation and function of dendritic cells and macrophages, down regulation of type-II IFN production, inhibition of NK cell cytotoxic activity, increased differentiation and IgA production by B cells, and prevention of proliferation and perforin/Fas ligand expression of CD8^+^ cytotoxic T cells [116].

Pulmonary viral infections can activate TGF-β, which then plays an important role in attenuating lung inflammation and immunopathology and promoting survival of the host [116,117]. Tregs produce TGF-β [118] and suppress effector functions of both innate and adaptive immune cells during influenza [78,79]. In addition, TGF-β is implicated in wound healing and tissue remodeling in the pulmonary tract through stimulation of matrix protein production and epithelial proliferation and differentiation [80,81]. Increased expression of TGF-β1 during acute asthma in mice confers resistance to IAV through suppression of tissue injury [82] but chronic overproduction of TGF-β can be detrimental and lead to development of fibrosis and thickening of the airway wall in humans [119]. This suggests that TGF-β1 could have therapeutic potential in preventing morbidity and mortality in acute infections such as early during an influenza pandemic before antigen-matched vaccines are available [82].

### 3.5. Lactoferrin

Lactoferrin is a multifunctional protein of the transferrin family and plays a vital role in disease tolerance in both plants and animals [120,121,122]. It is highly conserved with a molecular mass of about 80 kDa. It is widely found in most mammalian exocrine secretions, such as milk, saliva and tears, nasal and bronchial secretions, and intestinal secretions. Lactoferrin is also present in secondary granules of neutrophils and is secreted by some acinar cells [123]. The evidence suggests that lactoferrin acts as a first line of defense and plays a key role in normalization of immune homeostasis [121,124,125]. Lactoferrin was found to be a major component of innate immunity and to have an important role in control of acute septic inflammation and tissue damage [126,127,128]. By interacting with specific receptors on monocytes/macrophages and other cells, lactoferrin attenuates inflammation and contributes to tissue repair. Lactoferrin also protects against oxidative stress-induced cellular damage, in particular, by limiting production of hydroxyl radicals and lipid peroxidation [129]. In an animal model of tuberculosis, lactoferrin-treated mice showed reduced immunopathology and were able to modulate granulomatous formation without significant reduction in levels of cytokines (TNF-α, IL-1β, and IL-6) that are required to control infection [83,84]. Oral administration of lactoferrin to mice infected with virulent mycobacteria did not reduce pathogen numbers, but the severity of granuloma formation and lung pathology was ameliorated, leading to disease tolerance [85]. In acute and chronic lung infections caused by *Pseudomonas aeruginosa*, aerosolized lactoferrin resulted in non-significant reduction in bacterial loads but significant decreases in neutrophil recruitment and tissue damage. In addition, lactoferrin-treated mice recovered body weight more quickly and to a greater extent than untreated mice [86]. Lactoferrin can induce host tolerance and reduce immunopathology in other infections as well [87,88]. Lactoferrin can also protect against LPS-induced acute lung injury in mice [89].

### 3.6. Interferons (IFNs)

IFNs were the first cytokines described and include three well-characterized families [130]: type-I IFN produced by almost all cells in the body, type-II IFN produced by T cells, B cells, NK cells, and ILC1s, and type-III IFN produced mainly by stromal and epithelial cells [130]. These cytokines are known to be important for host defense against a variety of bacterial and viral pathogens [131,132], but can also cause pathological complications, depending on the infecting agent, the host, and the context of infection [132]. IAV initially causes inflammation in the nasal cavity by inducing type-I IFN, which then facilitates spread of colonizing or hospital-acquired bacteria to the lungs within the first few days of infection. The rapid replication of IAV in the lung epithelium leads to cell damage and exposure of the basement membrane, causing loss of barrier function that subsequently facilitates bacterial spread. Type-II IFN elaborated later in the IAV infection process alters the function and phagocytosis capacity of AM, which then fuels the growth of bacteria and uncontrolled tissue damage [43,50,90]. The continual replication of bacteria results in recruitment of inflammatory cells to the lung and weakens the repair process [50,90,91]. These results implicate host IFN responses during IAV infection in enhancement of secondary bacterial infection. While several groups have shown that virus-induced type-I IFN increases vulnerability to secondary bacterial infection [43,44,45,46,92] others, including our own group, have described a critical role for type-II IFN in mediating co-infection mortality during influenza [39,40,47,48,49]. Recently we showed that both type-I and type-II IFNs play complementary roles in mediating susceptibility to pneumococcal infection after IAV infection [50]. Type-I IFN is most important during early bacterial infection of the upper respiratory tract while type-II IFN inhibits bacterial clearance from the lower respiratory tract at later stages of infection. Therapeutic neutralization of the type-I IFN pathway early during co-infection combined with later neutralization of type-II IFN induces disease tolerance and improved survival of mice. These findings resolve the relative roles of type-1 versus type-II IFN during co-infection and suggest new therapeutic approaches for prevention of lethal bacterial superinfections in humans.

## 4. Innate Immune Cells in Disease Tolerance

There are several innate cells that are involved during early viral infection such as ILCs, AMs, neutrophils, and NK cells. ILCs are lymphocytes devoid of diversified antigen receptors expressed on T and B cells; they are tissue-resident cells, enriched at the mucosal surfaces of lungs, skin and intestine, and are activated by cytokines produced by tissue cells in response to infection or injury [133,134]. Transcriptional profiling of purified lung ILCs showed a correlation with genes involved of tissue healing, indicating an important role of ILCs in maintaining lung tissue homeostasis and disease tolerance [60]. Experimental evidence has indicated that ILC2s induce host disease tolerance during IAV infection through secretion of IL-5, IL-22 and AREG [60,71,76].

AMs are the major resident cell population that provides the first line of defense against lung pathogens. During IAV infection, AMs change their functions including lower phagocytic activity [40] and decreased patrolling between alveoli [41]. This in turn, leads to greater levels of inflammation and vascular leakage at barrier surfaces [135,136]. These effects can be mimicked by experimental ablation of AMs and reversed by exogenous replacement of AMs from uninfected mice [136]. AMs can also protect against RSV-induced lung damage [137]. It is thus not surprising that the loss of the protective efficacy of AMs during viral mono-infection can exacerbate pathology during secondary bacterial infection. Indeed, type-II IFN is a major mediator of these effects and neutralization of type-II IFN significantly increases survival to lethal pneumococcal co-infection following recovery from influenza [40]. However, AM function appears to have no effect on viral clearance mediated by adaptive immunity [138].

Neutrophils are the most abundant cell in the circulating innate immune system. There are reports of IAV-induced neutrophil dysfunction that is responsible for enhanced susceptibility to subsequent pneumococcus infection [51]. Exhaustion of neutrophils or treatment with anti-TRAIL (TNF-related apoptosis-inducing ligand) reduces bacterial outgrowth, leading to enhanced survival, indicating that these factors are involved in overactivation of neutrophils and diminished bacterial control during IAV—*Streptococcus pneumoniae* co-infection [139]. Similarly, during IAV-*Pseudomonas aeruginosa* co-infection, neutrophil dysfunction through decreased expression of G-CSF increases susceptibility to secondary bacterial infection [140]. Further studies indicated that increased neutrophilic influx during IAV infection mediates acute lung injury, which is attributable to alveolar capillary damage by neutrophil extracellular traps (NETs) [141]. Macrophage inflammatory protein 2 (MIP-2/CCL8) is known to attenuate neutrophil recruitment during IAV infection without reducing viral loads, eventually decreasing pathology [142]. Overall, while neutrophils can have protective functions including clearance of dead cells and viruses, elaboration of anti-microbial peptides, and interaction with protective macrophages, dendritic cells, and T cells, they can also have significant damaging effects through enhanced production of reactive oxygen, myeloperoxidase, matrix metalloproteinase, and NETs [36].

Another important innate cell is the NK cell that can kill virus-infected cells. NK cells respond to signals transmitted by infected respiratory epithelial cells in the early phases of virus replication. Lung resident NK cells play important roles in clearance of virus; however, they are also associated with severe tissue damage. During influenza, NK cells produce large amounts of type-II IFN that can cause acute lung damage and lethality [143,144]. Studies have shown that depletion of NK cells or neutralization of type-II IFN reduces mortality and improves tissue healing during IAV infection [143,145]. A similar observation was reported in the case of tularemia, in which mice devoid of NKT cells were better protected than mice with functional NKT cell populations [146]. An invariant NKT cell-macrophage immune axis causes chronic inflammation after viral infection due to continual stimulation of the innate immune response [147]. These various studies show that innate immune cells can have protective roles but also detrimental roles in modulating disease tolerance.

## 5. Impact of Microbiome, Aging, Obesity and Diabetes on Pulmonary Disease Tolerance

With the discovery of a community of bacteria, archaea, fungi, algae, and protists in the normal airways, the concept of a sterile lung is now outdated [148]. The unique and diverse lung microbiome is implicated in the maintenance of pulmonary health, a topic that has been extensively reviewed elsewhere [149,150,151,152]. The specific role of the microbiome in mediating disease tolerance, however, is not well understood and is a field poised for further discoveries. It is clear that following viral infection, normally colonizing bacteria can become pathogenic and cause secondary bacterial infections, leading to superinfection [50,153,154]. For instance, following influenza infection, the lung microbiome can be altered and dominated by drug resistant *Acinetobacter baumannii,* leading to secondary bacterial infections [155]. Influenza patients can also show increased growth of Leptotrichia, Oribacterium, Streptococcus, Atopobium, Eubacterium, Solobacterium and Rothia species in the respiratory tract. However, healthy individuals typically express only Haemophilus and Bacteroides species [156]. It has been demonstrated that intranasal administration of *Lactobacillus rhamnosus* can protect against respiratory syncytial virus (RSV) infection [157]. Further studies showed that the isolated peptidoglycan from *Lactobacillus rhamnosus* and commensal bacteria *Corynebacterium pseudodiphtheriticum* mediated improved resistance of infant mice to RSV and secondary pneumococcal infections [158,159,160]. Oral microbiota are found in the bronchoalveolar lavage fluid (BALF) of COVID-19 patients, which could pose a threat for co-infection [161]. In fact, *Moraxella catarrhalis* was found to be associated with SARS-CoV-2 co-infection, further suggesting a risk of co-infection with oral microbes following respiratory viral infection in these patients [162]. During influenza virus infection, the upregulation of type-I IFN causes inflammation in the upper respiratory tract and helps in translocation of colonizing bacteria to the lungs; deficiency in functional IFN-I pathways prevents co-infections [43,50]. Similar to bacteria, commensal fungi, protozoa, helminths, and viruses may have critical roles in disease tolerance but little is currently known about this.

Age is another important host factor that increases the risk of co-infection and decreases the rate of recovery from infection. Due to immunosenescence and decreased microbial diversity during aging, pathogens such as *S. pneumoniae* can establish chronic infections which become fatal following influenza infection [163]. In addition to aging, diabetes and obesity are important co-morbidities that may increase the probability of co-infection in SARS-CoV-2 patients [164]. Moreover, diabetes reduces the oral microbial diversity, leading to an increased risk of co-infections [165]. Overall, SARS-CoV-2 infection may allow commensal organisms in the oral cavity and pulmonary tract to enhance the cytokine storm, especially in aged patients and those with obesity, and diabetes [161].

## 6. Future Perspectives

The emergence of drug resistant bacteria [166,167], the inefficacy of antibiotics against post-influenza bacterial pneumonia [168] and the limited efficacy of bacterial vaccination in preventing influenza-*Streptococcus pneumoniae* co-infection [169], has necessitated development of alternative approaches that utilize disease tolerance for treatment of viral-bacterial co-infections. There are several mechanisms that the host engages during resolution of infection and inflammation as part of the healing process. Multiple cytokines and innate immune cells play essential roles in modulating the severe inflammatory process to repair and heal damaged tissues. This facilitates the return to a state of host homeostasis and therefore represents a promising therapeutic avenue for treatment of microbial co-infections. Additionally, for viruses such as SARS-CoV-2, elucidating disease tolerance mechanisms, for example in bats, which can be infected with numerous human pathogenic viruses but fail to develop clinical disease, may provide new therapeutic strategies for increasing human health.

## Figures and Tables

**Table 1:** Summary of cytokines that are involved in disease tolerance.

Model Host/Infection	Cytokine	Function	Reference
Dextran sodium sulfate-induced intestinal inflammation in mice	AREG	Reduced inflammation	[59]
IAV infection in mice	AREG	Epithelial integrity and tissue homeostasis	[60]
IAV-legionella co-infection in mice	AREG	Upregulation of genes involved in tissue healing	[61]
Dectin-deficient mice infected with Candida and Mycobacterium	AREG	Tissue repair	[62]
Parasitic helminth infection/*Trichuris muris* in mice	AREG	Clearance of parasites and tissue healing, suppression of inflammation	[63,64,65,66]
Enteric infection with Shigella, *E. coli*, Helicobacter, Neisseria in human tissue	AREG	Healing by transcriptional upregulation	[67,68,69,70]
IAV infection in mice	IL-5	Tissue integrity and host resistance	[71,72]
IAV-bacterial co-infection in mice	IL-22	Reduction in inflammation, tissue integrity, induce tolerance	[73,74]
IAV infection in mice	IL-22	Regeneration and repair of tissue	[75,76]
Ulcerative colitis in mice	IL-22	Regeneration and repair of tissue	[77]
IAV infection in mice	TGF-β	Attenuation of lung inflammation	[78,79]
Hyperoxic lung injury in mouse and rats	TGF-β	Epithelial repair	[80,81]
IAV infection in acute asthmatic mice	TGF-β	Suppression of tissue injury	[82]
Tuberculosis infection in mice	Lactoferrin	Reduce immunopathology	[83,84,85]
*Pseudomonas aeruginosa* lung infection	Lactoferrin	Tissue integrity	[86,87,88]
LPS-induced lung injury	Lactoferrin	Tissue integrity	[89]
Viral-bacterial co-infection in mice	IFNs	Tissue damage in the airways	[40,43,44,45,46,50,90,91,92]

## Data Availability

Not applicable.

## References

[1] Caldwell R.M., Schafer J.F., Compton L.E., Patterson F.L. (1958). Tolerance to cereal leaf rusts. Science.

[2] Schafer J.F. (1971). Tolerance to plant disease. Annu. Rev. Phytopathol..

[3] Painter R.H. (1958). Resistance of plants to insects. Annu. Rev. Entomol..

[4] Hilbert D.W., Swift D.M., Detling J.K., Dyer M.I. (1981). Relative growth rates and the grazing optimization hypothesis. Oecologia.

[5] Maschinski J., Whitham T.G. (1989). The continuum of plant responses to herbivory: The influence of plant association, nutrient availability, and timing. Am. Nat..

[6] Medzhitov R., Schneider D.S., Soares M.P. (2012). Disease tolerance as a defense strategy. Science.

[7] Raberg L., Sim D., Read A.F. (2007). Disentangling genetic variation for resistance and tolerance to infectious diseases in animals. Science.

[8] Schneider D.S., Ayres J.S. (2008). Two ways to survive infection: What resistance and tolerance can teach us about treating infectious diseases. Nat. Rev. Immunol..

[9] Ayres J.S., Freitag N., Schneider D.S. (2008). Identification of drosophila mutants altering defense of and endurance to Listeria monocytogenes infection. Genetics.

[10] Teixeira L., Ferreira A., Ashburner M. (2008). The bacterial symbiont Wolbachia induces resistance to RNA viral infections in Drosophila melanogaster. PLoS Biol..

[11] Gozzelino R., Andrade B.B., Larsen R., Luz N.F., Vanoaica L., Seixas E., Coutinho A., Cardoso S., Rebelo S., Poli M. (2012). Metabolic adaptation to tissue iron overload confers tolerance to malaria. Cell Host Microbe.

[12] Seixas E., Gozzelino R., Chora A., Ferreira A., Silva G., Larsen R., Rebelo S., Penido C., Smith N.R., Coutinho A. (2009). Heme oxygenase-1 affords protection against noncerebral forms of severe malaria. Proc. Natl. Acad. Sci. USA.

[13] Irving A.T., Ahn M., Goh G., Anderson D.E., Wang L.F. (2021). Lessons from the host defences of bats, a unique viral reservoir. Nature.

[14] Jebb D., Huang Z., Pippel M., Hughes G.M., Lavrichenko K., Devanna P., Winkler S., Jermiin L.S., Skirmuntt E.C., Katzourakis A. (2020). Six reference-quality genomes reveal evolution of bat adaptations. Nature.

[15] Teeling E.C., Springer M.S., Madsen O., Bates P., O’Brien S.J., Murphy W.J. (2005). A molecular phylogeny for bats illuminates biogeography and the fossil record. Science.

[16] Zhou P., Tachedjian M., Wynne J.W., Boyd V., Cui J., Smith I., Cowled C., Ng J.H., Mok L., Michalski W.P. (2016). Contraction of the type I IFN locus and unusual constitutive expression of IFN-alpha in bats. Proc. Natl. Acad. Sci. USA.

[17] De La Cruz-Rivera P.C., Kanchwala M., Liang H., Kumar A., Wang L.-F., Xing C., Schoggins J.W. (2018). The IFN response in bats displays distinctive IFN-stimulated gene expression kinetics with atypical RNASEL induction. J. Immunol..

[18] Koh J., Itahana Y., Mendenhall I.H., Low D., Soh E.X.Y., Guo A.K., Chionh Y.T., Wang L.F., Itahana K. (2019). ABCB1 protects bat cells from DNA damage induced by genotoxic compounds. Nat. Commun..

[19] Laing E.D., Sterling S.L., Weir D.L., Beauregard C.R., Smith I.L., Larsen S.E., Wang L.F., Snow A.L., Schaefer B.C., Broder C.C. (2019). Enhanced autophagy contributes to reduced viral infection in black flying fox cells. Viruses.

[20] Phillips A.M., Gonzalez L.O., Nekongo E.E., Ponomarenko A.I., McHugh S.M., Butty V.L., Levine S.S., Lin Y.S., Mirny L.A., Shoulders M.D. (2017). Host proteostasis modulates influenza evolution. Elife.

[21] Xie J., Li Y., Shen X., Goh G., Zhu Y., Cui J., Wang L.F., Shi Z.L., Zhou P. (2018). Dampened STING-dependent interferon activation in bats. Cell Host Microbe.

[22] Ahn M., Cui J., Irving A.T., Wang L.F. (2016). Unique loss of the PYHIN gene family in bats amongst mammals: Implications for inflammasome sensing. Sci. Rep..

[23] Wambua S., Mwangi T.W., Kortok M., Uyoga S.M., Macharia A.W., Mwacharo J.K., Weatherall D.J., Snow R.W., Marsh K., Williams T.N. (2006). The effect of alpha+-thalassaemia on the incidence of malaria and other diseases in children living on the coast of Kenya. PLoS Med..

[24] Albers G.A., Gray G.D., Piper L.R., Barker J.S., Le Jambre L.F., Barger I.A. (1987). The genetics of resistance and resilience to Haemonchus contortus infection in young merino sheep. Int. J. Parasitol..

[25] Hill A.V. (1998). The immunogenetics of human infectious diseases. Annu. Rev. Immunol..

[26] Malo D., Skamene E. (1994). Genetic control of host resistance to infection. Trends Genet..

[27] Smith J.A., Wilson K., Pilkington J.G., Pemberton J.M. (1999). Heritable variation in resistance to gastro-intestinal nematodes in an unmanaged mammal population. Proc. Biol. Sci..

[28] Soares M.P., Teixeira L., Moita L.F. (2017). Disease tolerance and immunity in host protection against infection. Nat. Rev. Immunol..

[29] Beadling C., Slifka M.K. (2004). How do viral infections predispose patients to bacterial infections?. Curr. Opin. Infect. Dis..

[30] Hament J.M., Kimpen J.L., Fleer A., Wolfs T.F. (1999). Respiratory viral infection predisposing for bacterial disease: A concise review. FEMS Immunol. Med. Microbiol..

[31] McCullers J.A. (2006). Insights into the interaction between influenza virus and pneumococcus. Clin. Microbiol. Rev..

[32] Morris D.E., Cleary D.W., Clarke S.C. (2017). Secondary bacterial infections associated with influenza pandemics. Front. Microbiol..

[33] Medzhitov R. (2010). Inflammation 2010: New adventures of an old flame. Cell.

[34] Nathan C., Ding A. (2010). Nonresolving inflammation. Cell.

[35] Chertov O., Yang D., Howard O.M., Oppenheim J.J. (2000). Leukocyte granule proteins mobilize innate host defenses and adaptive immune responses. Immunol. Rev..

[36] Johansson C., Kirsebom F.C.M. (2021). Neutrophils in respiratory viral infections. Mucosal Immunol..

[37] Nathan C. (2006). Neutrophils and immunity: Challenges and opportunities. Nat. Rev. Immunol..

[38] Iwasaki A., Pillai P.S. (2014). Innate immunity to influenza virus infection. Nat. Rev. Immunol..

[39] Califano D., Furuya Y., Metzger D.W. (2018). Effects of influenza on alveolar macrophage viability are dependent on mouse genetic strain. J. Immunol..

[40] Sun K., Metzger D.W. (2008). Inhibition of pulmonary antibacterial defense by interferon-gamma during recovery from influenza infection. Nat. Med..

[41] Neupane A.S., Willson M., Chojnacki A.K., Vargas E.S.C.F., Morehouse C., Carestia A., Keller A.E., Peiseler M., DiGiandomenico A., Kelly M.M. (2020). Patrolling alveolar macrophages conceal bacteria from the immune system to maintain homeostasis. Cell.

[42] Aguilera E.R., Lenz L.L. (2020). Inflammation as a modulator of host susceptibility to pulmonary influenza, pneumococcal, and co-Infections. Front. Immunol..

[43] Nakamura S., Davis K.M., Weiser J.N. (2011). Synergistic stimulation of type I interferons during influenza virus coinfection promotes Streptococcus pneumoniae colonization in mice. J. Clin. Investig..

[44] Shahangian A., Chow E.K., Tian X., Kang J.R., Ghaffari A., Liu S.Y., Belperio J.A., Cheng G., Deng J.C. (2009). Type I IFNs mediate development of postinfluenza bacterial pneumonia in mice. J. Clin. Investig..

[45] Shepardson K.M., Larson K., Morton R.V., Prigge J.R., Schmidt E.E., Huber V.C., Rynda-Apple A. (2016). Differential type I interferon signaling is a master regulator of susceptibility to postinfluenza bacterial superinfection. mBio.

[46] Shirey K.A., Perkins D.J., Lai W., Zhang W., Fernando L.R., Gusovsky F., Blanco J.C.G., Vogel S.N. (2019). Influenza “trains” the host for enhanced susceptibility to secondary bacterial infection. mBio.

[47] Breslow-Deckman J.M., Mattingly C.M., Birket S.E., Hoskins S.N., Ho T.N., Garvy B.A., Feola D.J. (2013). Linezolid decreases susceptibility to secondary bacterial pneumonia postinfluenza infection in mice through its effects on IFN-gamma. J. Immunol..

[48] Hang D.T.T., Choi E.-J., Song J.-Y., Kim S.-E., Kwak J., Shin Y.-K. (2011). Differential effect of prior influenza infection on alveolar macrophage phagocytosis of Staphylococcus aureus and Escherichia coli: Involvement of interferon-gamma production. Microbiol. Immunol..

[49] Sun K., Ye J., Perez D.R., Metzger D.W. (2011). Seasonal FluMist vaccination induces cross-reactive T cell immunity against H1N1 (2009) influenza and secondary bacterial infections. J. Immunol..

[50] Barman T.K., Racine R., Bonin J.L., Califano D., Salmon S.L., Metzger D.W. (2021). Sequential targeting of interferon pathways for increased host resistance to bacterial superinfection during influenza. PLoS Pathog..

[51] McNamee L.A., Harmsen A.G. (2006). Both influenza-induced neutrophil dysfunction and neutrophil-independent mechanisms contribute to increased susceptibility to a secondary Streptococcus pneumoniae infection. Infect. Immun..

[52] Wang X., Yuan J., Wang H., Gan N., Zhang Q., Liu B., Wang J., Shu Z., Rao L., Gou X. (2019). Progranulin decreases susceptibility to streptococcus pneumoniae in influenza and protects against lethal coinfection. J. Immunol..

[53] Didierlaurent A., Goulding J., Patel S., Snelgrove R., Low L., Bebien M., Lawrence T., van Rijt L.S., Lambrecht B.N., Sirard J.C. (2008). Sustained desensitization to bacterial Toll-like receptor ligands after resolution of respiratory influenza infection. J. Exp. Med..

[54] Kohler J., Breitbach K., Renner C., Heitsch A.K., Bast A., van Rooijen N., Vogelgesang S., Steinmetz I. (2011). NADPH-oxidase but not inducible nitric oxide synthase contributes to resistance in a murine Staphylococcus aureus Newman pneumonia model. Microbes Infect..

[55] van der Sluijs K.F., Nijhuis M., Levels J.H., Florquin S., Mellor A.L., Jansen H.M., van der Poll T., Lutter R. (2006). Influenza-induced expression of indoleamine 2,3-dioxygenase enhances interleukin-10 production and bacterial outgrowth during secondary pneumococcal pneumonia. J. Infect. Dis..

[56] Lijek R.S., Weiser J.N. (2012). Co-infection subverts mucosal immunity in the upper respiratory tract. Curr. Opin. Immunol..

[57] D’Elia R.V., Harrison K., Oyston P.C., Lukaszewski R.A., Clark G.C. (2013). Targeting the “cytokine storm” for therapeutic benefit. Clin. Vaccine Immunol..

[58] Read A.F., Graham A.L., Raberg L. (2008). Animal defenses against infectious agents: Is damage control more important than pathogen control. PLoS Biol..

[59] Monticelli L.A., Osborne L.C., Noti M., Tran S.V., Zaiss D.M., Artis D. (2015). IL-33 promotes an innate immune pathway of intestinal tissue protection dependent on amphiregulin-EGFR interactions. Proc. Natl. Acad. Sci. USA.

[60] Monticelli L.A., Sonnenberg G.F., Abt M.C., Alenghat T., Ziegler C.G., Doering T.A., Angelosanto J.M., Laidlaw B.J., Yang C.Y., Sathaliyawala T. (2011). Innate lymphoid cells promote lung-tissue homeostasis after infection with influenza virus. Nat. Immunol..

[61] Jamieson A.M., Pasman L., Yu S., Gamradt P., Homer R.J., Decker T., Medzhitov R. (2013). Role of tissue protection in lethal respiratory viral-bacterial coinfection. Science.

[62] Branzk N., Lubojemska A., Hardison S.E., Wang Q., Gutierrez M.G., Brown G.D., Papayannopoulos V. (2014). Neutrophils sense microbe size and selectively release neutrophil extracellular traps in response to large pathogens. Nat. Immunol..

[63] Gause W.C., Wynn T.A., Allen J.E. (2013). Type 2 immunity and wound healing: Evolutionary refinement of adaptive immunity by helminths. Nat. Rev. Immunol..

[64] Pulendran B., Artis D. (2012). New paradigms in type 2 immunity. Science.

[65] Anthony R.M., Rutitzky L.I., Urban J.F., Stadecker M.J., Gause W.C. (2007). Protective immune mechanisms in helminth infection. Nat. Rev. Immunol..

[66] Zaiss D.M., Yang L., Shah P.R., Kobie J.J., Urban J.F., Mosmann T.R. (2006). Amphiregulin, a TH2 cytokine enhancing resistance to nematodes. Science.

[67] Busiello I., Acquaviva R., Di Popolo A., Blanchard T.G., Ricci V., Romano M., Zarrilli R. (2004). Helicobacter pylori gamma-glutamyltranspeptidase upregulates COX-2 and EGF-related peptide expression in human gastric cells. Cell Microbiol..

[68] Kim Y., Oh S., Park S., Kim S.H. (2009). Interactive transcriptome analysis of enterohemorrhagic Escherichia coli (EHEC) O157:H7 and intestinal epithelial HT-29 cells after bacterial attachment. Int. J. Food Microbiol..

[69] Lofmark S., de Klerk N., Aro H. (2011). Neisseria gonorrhoeae infection induces altered amphiregulin processing and release. PLoS ONE.

[70] Pedron T., Thibault C., Sansonetti P.J. (2003). The invasive phenotype of Shigella flexneri directs a distinct gene expression pattern in the human intestinal epithelial cell line Caco-2. J. Biol. Chem..

[71] Califano D., Furuya Y., Roberts S., Avram D., McKenzie A.N.J., Metzger D.W. (2018). IFN-gamma increases susceptibility to influenza A infection through suppression of group II innate lymphoid cells. Mucosal Immunol..

[72] Rothenberg M.E., Hogan S.P. (2006). The eosinophil. Annu. Rev. Immunol..

[73] Ivanov S., Renneson J., Fontaine J., Barthelemy A., Paget C., Fernandez E.M., Blanc F., De Trez C., Van Maele L., Dumoutier L. (2013). Interleukin-22 reduces lung inflammation during influenza A virus infection and protects against secondary bacterial infection. J. Virol..

[74] Barthelemy A., Sencio V., Soulard D., Deruyter L., Faveeuw C., Le Goffic R., Trottein F. (2018). Interleukin-22 immunotherapy during severe influenza enhances lung tissue integrity and reduces secondary bacterial systemic invasion. Infect. Immun..

[75] Kumar P., Thakar M.S., Ouyang W., Malarkannan S. (2013). IL-22 from conventional NK cells is epithelial regenerative and inflammation protective during influenza infection. Mucosal Immunol..

[76] Pociask D.A., Scheller E.V., Mandalapu S., McHugh K.J., Enelow R.I., Fattman C.L., Kolls J.K., Alcorn J.F. (2013). IL-22 is essential for lung epithelial repair following influenza infection. Am. J. Pathol..

[77] Sugimoto K., Ogawa A., Mizoguchi E., Shimomura Y., Andoh A., Bhan A.K., Blumberg R.S., Xavier R.J., Mizoguchi A. (2008). IL-22 ameliorates intestinal inflammation in a mouse model of ulcerative colitis. J. Clin. Investig..

[78] Antunes I., Kassiotis G. (2010). Suppression of innate immune pathology by regulatory T cells during Influenza A virus infection of immunodeficient mice. J. Virol..

[79] Betts R.J., Prabhu N., Ho A.W., Lew F.C., Hutchinson P.E., Rotzschke O., Macary P.A., Kemeny D.M. (2012). Influenza A virus infection results in a robust, antigen-responsive, and widely disseminated Foxp3+ regulatory T cell response. J. Virol..

[80] Buckley S., Shi W., Barsky L., Warburton D. (2008). TGF-beta signaling promotes survival and repair in rat alveolar epithelial type 2 cells during recovery after hyperoxic injury. Am. J. Physiol. Lung Cell. Mol. Physiol..

[81] Crosby L.M., Waters C.M. (2010). Epithelial repair mechanisms in the lung. Am. J. Physiol. Lung Cell. Mol. Physiol..

[82] Furuya Y., Furuya A.K., Roberts S., Sanfilippo A.M., Salmon S.L., Metzger D.W. (2015). Prevention of influenza virus-induced immunopathology by TGF-beta produced during allergic asthma. PLoS Pathog..

[83] Actor J.K. (2015). Lactoferrin: A modulator for immunity against tuberculosis related granulomatous pathology. Mediat. Inflamm..

[84] Welsh K.J., Hwang S.A., Hunter R.L., Kruzel M.L., Actor J.K. (2010). Lactoferrin modulation of mycobacterial cord factor trehalose 6-6′-dimycolate induced granulomatous response. Transl. Res..

[85] Welsh K.J., Hwang S.A., Boyd S., Kruzel M.L., Hunter R.L., Actor J.K. (2011). Influence of oral lactoferrin on Mycobacterium tuberculosis induced immunopathology. Tuberculosis (Edinb.).

[86] Valenti P., Frioni A., Rossi A., Ranucci S., De Fino I., Cutone A., Rosa L., Bragonzi A., Berlutti F. (2017). Aerosolized bovine lactoferrin reduces neutrophils and pro-inflammatory cytokines in mouse models of Pseudomonas aeruginosa lung infections. Biochem. Cell Biol..

[87] Legrand D. (2012). Lactoferrin, a key molecule in immune and inflammatory processes. Biochem. Cell Biol..

[88] Valenti P., Catizone A., Pantanella F., Frioni A., Natalizi T., Tendini M., Berlutti F. (2011). Lactoferrin decreases inflammatory response by cystic fibrosis bronchial cells invaded with Burkholderia cenocepacia iron-modulated biofilm. Int. J. Immunopathol. Pharm..

[89] Li X.J., Liu D.P., Chen H.L., Pan X.H., Kong Q.Y., Pang Q.F. (2012). Lactoferrin protects against lipopolysaccharide-induced acute lung injury in mice. Int. Immunopharmacol..

[90] Chertow D.S., Memoli M.J. (2013). Bacterial coinfection in influenza: A grand rounds review. JAMA.

[91] Cayrol C., Girard J.P. (2014). IL-33: An alarmin cytokine with crucial roles in innate immunity, inflammation and allergy. Curr. Opin. Immunol..

[92] Lee B., Robinson K.M., McHugh K.J., Scheller E.V., Mandalapu S., Chen C., Di Y.P., Clay M.E., Enelow R.I., Dubin P.J. (2015). Influenza-induced type I interferon enhances susceptibility to gram-negative and gram-positive bacterial pneumonia in mice. Am. J. Physiol.-Lung Cell Mol. Physiol..

[93] Shoyab M., Plowman G.D., McDonald V.L., Bradley J.G., Todaro G.J. (1989). Structure and function of human amphiregulin: A member of the epidermal growth factor family. Science.

[94] Mograbi B., Rochet N., Imbert V., Bourget I., Bocciardi R., Emiliozzi C., Rossi B. (1997). Human monocytes express amphiregulin and heregulin growth factors upon activation. Eur. Cytokine Netw..

[95] Zaiss D.M.W., Gause W.C., Osborne L.C., Artis D. (2015). Emerging functions of amphiregulin in orchestrating immunity, inflammation, and tissue repair. Immunity.

[96] Plowman G.D., Green J.M., McDonald V.L., Neubauer M.G., Disteche C.M., Todaro G.J., Shoyab M. (1990). The amphiregulin gene encodes a novel epidermal growth factor-related protein with tumor-inhibitory activity. Mol. Cell Biol..

[97] Stern K.A., Place T.L., Lill N.L. (2008). EGF and amphiregulin differentially regulate Cbl recruitment to endosomes and EGF receptor fate. Biochem. J..

[98] Takeuchi S., Yano S. (2014). Clinical significance of epidermal growth factor receptor tyrosine kinase inhibitors: Sensitivity and resistance. Respir. Investig..

[99] Lee J.S., Campbell H.D., Kozak C.A., Young I.G. (1989). The IL-4 and IL-5 genes are closely linked and are part of a cytokine gene cluster on mouse chromosome 11. Somat. Cell Mol. Genet..

[100] Milburn M.V., Hassell A.M., Lambert M.H., Jordan S.R., Proudfoot A.E., Graber P., Wells T.N. (1993). A novel dimer configuration revealed by the crystal structure at 2.4 A resolution of human interleukin-5. Nature.

[101] van Leeuwen B.H., Martinson M.E., Webb G.C., Young I.G. (1989). Molecular organization of the cytokine gene cluster, involving the human IL-3, IL-4, IL-5, and GM-CSF genes, on human chromosome 5. Blood.

[102] Kaminuma O., Mori A., Kitamura N., Hashimoto T., Kitamura F., Inokuma S., Miyatake S. (2005). Role of GATA-3 in IL-5 gene transcription by CD4+ T cells of asthmatic patients. Int. Arch. Allergy Immunol..

[103] Bradding P., Roberts J.A., Britten K.M., Montefort S., Djukanovic R., Mueller R., Heusser C.H., Howarth P.H., Holgate S.T. (1994). Interleukin-4, -5, and -6 and tumor necrosis factor-alpha in normal and asthmatic airways: Evidence for the human mast cell as a source of these cytokines. Am. J. Respir. Cell Mol. Biol..

[104] Dubucquoi S., Desreumaux P., Janin A., Klein O., Goldman M., Tavernier J., Capron A., Capron M. (1994). Interleukin 5 synthesis by eosinophils: Association with granules and immunoglobulin-dependent secretion. J. Exp. Med..

[105] Takatsu K. (2011). Interleukin-5 and IL-5 receptor in health and diseases. Proc. Jpn. Acad Ser. B Phys. Biol Sci.

[106] Gorski S.A., Hahn Y.S., Braciale T.J. (2013). Group 2 innate lymphoid cell production of IL-5 is regulated by NKT cells during influenza virus infection. PLoS Pathog..

[107] Hufford M.M., Kim T.S., Sun J., Braciale T.J. (2011). Antiviral CD8+ T cell effector activities in situ are regulated by target cell type. J. Exp. Med..

[108] Spits H., Artis D., Colonna M., Diefenbach A., Di Santo J.P., Eberl G., Koyasu S., Locksley R.M., McKenzie A.N., Mebius R.E. (2013). Innate lymphoid cells—A proposal for uniform nomenclature. Nat. Rev. Immunol..

[109] Sonnenberg G.F., Fouser L.A., Artis D. (2011). Border patrol: Regulation of immunity, inflammation and tissue homeostasis at barrier surfaces by IL-22. Nat. Immunol..

[110] Dudakov J.A., Hanash A.M., van den Brink M.R. (2015). Interleukin-22: Immunobiology and pathology. Annu. Rev. Immunol..

[111] Muhl H., Scheiermann P., Bachmann M., Hardle L., Heinrichs A., Pfeilschifter J. (2013). IL-22 in tissue-protective therapy. Br. J. Pharm..

[112] Sabat R., Ouyang W., Wolk K. (2014). Therapeutic opportunities of the IL-22-IL-22R1 system. Nat. Rev. Drug Discov..

[113] Kulkarni A.B., Huh C.G., Becker D., Geiser A., Lyght M., Flanders K.C., Roberts A.B., Sporn M.B., Ward J.M., Karlsson S. (1993). Transforming growth factor beta 1 null mutation in mice causes excessive inflammatory response and early death. Proc. Natl. Acad. Sci. USA.

[114] Letterio J.J., Roberts A.B. (1996). Transforming growth factor-beta1-deficient mice: Identification of isoform-specific activities in vivo. J. Leukoc. Biol..

[115] Shull M.M., Ormsby I., Kier A.B., Pawlowski S., Diebold R.J., Yin M., Allen R., Sidman C., Proetzel G., Calvin D. (1992). Targeted disruption of the mouse transforming growth factor-beta 1 gene results in multifocal inflammatory disease. Nature.

[116] Williams A.E., Humphreys I.R., Cornere M., Edwards L., Rae A., Hussell T. (2005). TGF-beta prevents eosinophilic lung disease but impairs pathogen clearance. Microbes Infect..

[117] Carlson C.M., Turpin E.A., Moser L.A., O’Brien K.B., Cline T.D., Jones J.C., Tumpey T.M., Katz J.M., Kelley L.A., Gauldie J. (2010). Transforming growth factor-beta: Activation by neuraminidase and role in highly pathogenic H5N1 influenza pathogenesis. PLoS Pathog..

[118] D’Alessio F.R., Tsushima K., Aggarwal N.R., West E.E., Willett M.H., Britos M.F., Pipeling M.R., Brower R.G., Tuder R.M., McDyer J.F. (2009). CD4+CD25+Foxp3+ Tregs resolve experimental lung injury in mice and are present in humans with acute lung injury. J. Clin. Investig..

[119] Yang Y.C., Zhang N., Van Crombruggen K., Hu G.H., Hong S.L., Bachert C. (2012). Transforming growth factor-beta1 in inflammatory airway disease: A key for understanding inflammation and remodeling. Allergy.

[120] Malnoy M., Venisse J.-S., Brisset M.-N., Chevreau E. (2003). Expression of bovine lactoferrin cDNA confers resistance to Erwinia amylovora in transgenic pear. Mol. Breed..

[121] Sanchez L., Calvo M., Brock J.H. (1992). Biological role of lactoferrin. Arch. Dis. Child..

[122] Zhang Z., Coyne D.P., Vidaver A.K., Mitra A. (1998). Expression of human lactoferrin cDNA confers resistance to ralstonia solanacearum in transgenic tobacco plants. Phytopathology.

[123] Actor J.K., Hwang S.A., Kruzel M.L. (2009). Lactoferrin as a natural immune modulator. Curr. Pharm. Des..

[124] Kruzel M.L., Actor J.K., Boldogh I., Zimecki M. (2007). Lactoferrin in health and disease. Postepy Hig. Med. Dosw. (Online).

[125] Lonnerdal B., Iyer S. (1995). Lactoferrin: Molecular structure and biological function. Annu. Rev. Nutr..

[126] Baveye S., Elass E., Mazurier J., Spik G., Legrand D. (1999). Lactoferrin: A multifunctional glycoprotein involved in the modulation of the inflammatory process. Clin. Chem. Lab. Med..

[127] Kruzel M.L., Harari Y., Mailman D., Actor J.K., Zimecki M. (2002). Differential effects of prophylactic, concurrent and therapeutic lactoferrin treatment on LPS-induced inflammatory responses in mice. Clin. Exp. Immunol..

[128] Ochoa T.J., Chea-Woo E., Baiocchi N., Pecho I., Campos M., Prada A., Valdiviezo G., Lluque A., Lai D., Cleary T.G. (2013). Randomized double-blind controlled trial of bovine lactoferrin for prevention of diarrhea in children. J. Pediatr..

[129] Kruzel M.L., Zimecki M., Actor J.K. (2017). Lactoferrin in a context of inflammation-induced pathology. Front. Immunol..

[130] Pestka S., Krause C.D., Walter M.R. (2004). Interferons, interferon-like cytokines, and their receptors. Immunol. Rev..

[131] Gonzales-van Horn S.R., Farrar J.D. (2015). Interferon at the crossroads of allergy and viral infections. J. Leukoc. Biol..

[132] McNab F., Mayer-Barber K., Sher A., Wack A., O’Garra A. (2015). Type I interferons in infectious disease. Nat. Rev. Immunol..

[133] Morita H., Moro K., Koyasu S. (2016). Innate lymphoid cells in allergic and nonallergic inflammation. J. Allergy Clin. Immunol..

[134] Vivier E., Artis D., Colonna M., Diefenbach A., Di Santo J.P., Eberl G., Koyasu S., Locksley R.M., McKenzie A.N.J., Mebius R.E. (2018). Innate lymphoid cells: 10 years on. Cell.

[135] Johnston L.K., Rims C.R., Gill S.E., McGuire J.K., Manicone A.M. (2012). Pulmonary macrophage subpopulations in the induction and resolution of acute lung injury. Am. J. Respir. Cell Mol. Biol..

[136] Purnama C., Ng S.L., Tetlak P., Setiagani Y.A., Kandasamy M., Baalasubramanian S., Karjalainen K., Ruedl C. (2014). Transient ablation of alveolar macrophages leads to massive pathology of influenza infection without affecting cellular adaptive immunity. Eur. J. Immunol..

[137] Kolli D., Gupta M.R., Sbrana E., Velayutham T.S., Chao H., Casola A., Garofalo R.P. (2014). Alveolar macrophages contribute to the pathogenesis of human metapneumovirus infection while protecting against respiratory syncytial virus infection. Am. J. Respir. Cell Mol. Biol..

[138] Schneider C., Nobs S.P., Heer A.K., Kurrer M., Klinke G., van Rooijen N., Vogel J., Kopf M. (2014). Alveolar macrophages are essential for protection from respiratory failure and associated morbidity following influenza virus infection. PLoS Pathog..

[139] Ellis G.T., Davidson S., Crotta S., Branzk N., Papayannopoulos V., Wack A. (2015). TRAIL+ monocytes and monocyte-related cells cause lung damage and thereby increase susceptibility to influenza-Streptococcus pneumoniae coinfection. EMBO Rep..

[140] Ishikawa H., Fukui T., Ino S., Sasaki H., Awano N., Kohda C., Tanaka K. (2016). Influenza virus infection causes neutrophil dysfunction through reduced G-CSF production and an increased risk of secondary bacteria infection in the lung. Virology.

[141] Narasaraju T., Yang E., Samy R.P., Ng H.H., Poh W.P., Liew A.A., Phoon M.C., van Rooijen N., Chow V.T. (2011). Excessive neutrophils and neutrophil extracellular traps contribute to acute lung injury of influenza pneumonitis. Am. J. Pathol..

[142] Sakai S., Kawamata H., Mantani N., Kogure T., Shimada Y., Terasawa K., Sakai T., Imanishi N., Ochiai H. (2000). Therapeutic effect of anti-macrophage inflammatory protein 2 antibody on influenza virus-induced pneumonia in mice. J. Virol..

[143] Abdul-Careem M.F., Mian M.F., Yue G., Gillgrass A., Chenoweth M.J., Barra N.G., Chew M.V., Chan T., Al-Garawi A.A., Jordana M. (2012). Critical role of natural killer cells in lung immunopathology during influenza infection in mice. J. Infect. Dis..

[144] Culley F.J. (2009). Natural killer cells in infection and inflammation of the lung. Immunology.

[145] Li H., Singh S., Potula R., Persidsky Y., Kanmogne G.D. (2014). Dysregulation of claudin-5 in HIV-induced interstitial pneumonitis and lung vascular injury. Protective role of peroxisome proliferator-activated receptor-gamma. Am. J. Respir. Crit. Care Med..

[146] Hill T.M., Gilchuk P., Cicek B.B., Osina M.A., Boyd K.L., Durrant D.M., Metzger D.W., Khanna K.M., Joyce S. (2015). Border patrol gone awry: Lung NKT cell activation by Francisella tularensis exacerbates tularemia-like disease. PLoS Pathog..

[147] Kim E.Y., Battaile J.T., Patel A.C., You Y., Agapov E., Grayson M.H., Benoit L.A., Byers D.E., Alevy Y., Tucker J. (2008). Persistent activation of an innate immune response translates respiratory viral infection into chronic lung disease. Nat. Med..

[148] Marchesi J.R., Ravel J. (2015). The vocabulary of microbiome research: A proposal. Microbiome.

[149] Budden K.F., Gellatly S.L., Wood D.L., Cooper M.A., Morrison M., Hugenholtz P., Hansbro P.M. (2017). Emerging pathogenic links between microbiota and the gut-lung axis. Nat. Rev. Microbiol..

[150] Ichinohe T., Pang I.K., Kumamoto Y., Peaper D.R., Ho J.H., Murray T.S., Iwasaki A. (2011). Microbiota regulates immune defense against respiratory tract influenza A virus infection. Proc. Natl. Acad. Sci. USA.

[151] Marsland B.J., Gollwitzer E.S. (2014). Host-microorganism interactions in lung diseases. Nat. Rev. Immunol..

[152] Wypych T.P., Wickramasinghe L.C., Marsland B.J. (2019). The influence of the microbiome on respiratory health. Nat. Immunol..

[153] Bellinghausen C., Gulraiz F., Heinzmann A.C., Dentener M.A., Savelkoul P.H., Wouters E.F., Rohde G.G., Stassen F.R. (2016). Exposure to common respiratory bacteria alters the airway epithelial response to subsequent viral infection. Respir. Res..

[154] Dickson R.P., Singer B.H., Newstead M.W., Falkowski N.R., Erb-Downward J.R., Standiford T.J., Huffnagle G.B. (2016). Enrichment of the lung microbiome with gut bacteria in sepsis and the acute respiratory distress syndrome. Nat. Microbiol..

[155] Hu Y., Zhang Y., Ren X., Liu Y., Xiao Y., Li L., Yang F., Su H., Liu F., Liu H. (2016). A case report demonstrating the utility of next generation sequencing in analyzing serial samples from the lung following an infection with influenza A (H7N9) virus. J. Clin. Virol..

[156] Lu H.F., Li A., Zhang T., Ren Z.G., He K.X., Zhang H., Yang J.Z., Luo Q.X., Zhou K., Chen C.L. (2017). Disordered oropharyngeal microbial communities in H7N9 patients with or without secondary bacterial lung infection. Emerg. Microbes Infect..

[157] Tomosada Y., Chiba E., Zelaya H., Takahashi T., Tsukida K., Kitazawa H., Alvarez S., Villena J. (2013). Nasally administered Lactobacillus rhamnosus strains differentially modulate respiratory antiviral immune responses and induce protection against respiratory syncytial virus infection. BMC Immunol..

[158] Clua P., Kanmani P., Zelaya H., Tada A., Kober A., Salva S., Alvarez S., Kitazawa H., Villena J. (2017). Peptidoglycan from immunobiotic Lactobacillus rhamnosus improves resistance of infant mice to respiratory syncytial viral Infection and secondary pneumococcal pneumonia. Front. Immunol..

[159] Clua P., Tomokiyo M., Raya Tonetti F., Islam M.A., Garcia Castillo V., Marcial G., Salva S., Alvarez S., Takahashi H., Kurata S. (2020). The role of alveolar macrophages in the improved protection against respiratory syncytial virus and pneumococcal superinfection induced by the peptidoglycan of Lactobacillus rhamnosus CRL1505. Cells.

[160] Kanmani P., Clua P., Vizoso-Pinto M.G., Rodriguez C., Alvarez S., Melnikov V., Takahashi H., Kitazawa H., Villena J. (2017). Respiratory commensal bacteria Corynebacterium pseudodiphtheriticum improves resistance of infant mice to respiratory syncytial virus and Streptococcus pneumoniae superinfection. Front. Microbiol..

[161] Bao L., Zhang C., Dong J., Zhao L., Li Y., Sun J. (2020). Oral microbiome and SARS-CoV-2: Beware of lung co-infection. Front. Microbiol..

[162] Peddu V., Shean R.C., Xie H., Shrestha L., Perchetti G.A., Minot S.S., Roychoudhury P., Huang M.L., Nalla A., Reddy S.B. (2020). Metagenomic analysis reveals clinical SARS-CoV-2 infection and bacterial or viral superinfection and colonization. Clin. Chem..

[163] Krone C.L., van de Groep K., Trzcinski K., Sanders E.A., Bogaert D. (2014). Immunosenescence and pneumococcal disease: An imbalance in host-pathogen interactions. Lancet Respir. Med..

[164] Alosaimi B., Naeem A., Hamed M.E., Alkadi H.S., Alanazi T., Al Rehily S.S., Almutairi A.Z., Zafar A. (2021). Influenza co-infection associated with severity and mortality in COVID-19 patients. Virol. J..

[165] Saeb A.T.M., Al-Rubeaan K.A., Aldosary K., Udaya Raja G.K., Mani B., Abouelhoda M., Tayeb H.T. (2019). Relative reduction of biological and phylogenetic diversity of the oral microbiota of diabetes and pre-diabetes patients. Microb. Pathog..

[166] Gould K. (2016). Antibiotics: From prehistory to the present day. J. Antimicrob. Chemother..

[167] Neu H.C. (1992). The crisis in antibiotic resistance. Science.

[168] Sun K., Yajjala V.K., Bauer C., Talmon G.A., Fischer K.J., Kielian T., Metzger D.W. (2016). Nox2-derived oxidative stress results in inefficacy of antibiotics against post-influenza S. aureus pneumonia. J. Exp. Med..

[169] Metzger D.W., Furuya Y., Salmon S.L., Roberts S., Sun K. (2015). Limited efficacy of antibacterial vaccination against secondary serotype 3 pneumococcal pneumonia following influenza infection. J. Infect. Dis..

